# Use of heated tobacco products by people with chronic diseases: The 2019 JASTIS study

**DOI:** 10.1371/journal.pone.0260154

**Published:** 2021-11-18

**Authors:** Chikako Nakama, Takahiro Tabuchi

**Affiliations:** 1 Department of Hygiene and Public Health, Kansai Medical University, Hirakata-shi, Osaka, Japan; 2 Cancer Control Center, Osaka International Cancer Institute, Osaka-shi, Osaka, Japan; Universite de Bretagne Occidentale, FRANCE

## Abstract

Heated tobacco products (HTPs) have become popular recently. People with chronic disease, such as diabetes, cardiovascular disease (CVD), chronic obstructive pulmonary disease (COPD) and cancer, should quit smoking for treatment and recurrence of tobacco-related diseases. However, they have difficulty in quitting smoking, and they may start HTPs use to quit smoking. The purpose of this study is to examine the use of HTPs in people with chronic disease. We used data from an internet study, the Japan Society and New Tobacco Internet Survey (JASTIS). We analyzed 9,008 respondents aged 15–73 years in 2019 using logistic regression. Current use of tobacco products was defined as use within the previous 30 days. Prevalence of current HTP use including dual use and dual use with cigarettes was 9.0% and 6.1% respectively in total. By disease: hypertension 10.2% and 7.4%, diabetes 15.9% and 12.3%, CVD 19.2% and 15.7%, COPD 40.5% and 33.3%, and cancer 17.5% and 11.9%. Diabetes, CVD, COPD, and cancer were positively associated with current use of HTPs (odds ratios (ORs) and 95% confidence intervals (CIs): 1.48 (1.06, 2.07), 2.29 (1.38, 3.80), 3.97(1.73, 9.11), and 3.58(1.99, 6.44), respectively) and dual use of cigarettes and HTPs (ORs and 95% CIs: 2.23 (1.61, 3.09), 3.58 (2.29, 5.60), 7.46 (3.76, 14.80), and 2.57 (1.46, 4.55), respectively) after adjusting for confounders. People with chronic disease were more likely to use HTPs and HTPs together with cigarettes. Further research on the smoking situation of HTPs in patients with chronic diseases is necessary.

## Introduction

Heated tobacco products (HTPs) are relatively new tobacco products, and have recently been taken up by smokers around the world as well as electronic cigarettes (e-cigarettes). While e-cigarettes have become popular in North America and Europe [1], in Japan, e-cigarettes with nicotine liquid have been prohibited by the Pharmaceutical Affairs Act., and HTPs has been expanding after IQOS (Philip Morris International Inc.) appeared on the Japanese market in 2014. In a recent internet survey in Japan, 22.1% of adults were current conventional cigarette smokers, and 6.6% had ever used HTPs (IQOS, Ploom TECH, and glo) or e-cigarettes; 1.3% had used them in the previous 30 days in 2015 [2]. The 2018 National Health and Nutrition Survey in Japan reported that half of 20–39 year-old male smokers and 40% of 30–39 year-old female smokers use HTPs [3].

It is known that some patients with chronic disease cannot stop smoking, and many patients who have to stop smoking for hospitalization and surgery, start smoking again as soon as they can. For example, a review reported that, only a third to a half of the smokers who suffer from myocardial infarction (MI) subsequently reduce or stop smoking [4]. Also, it has been reported that the self-reported prevalence of smoking among cancer survivors ranges between 9% and 33%, and even if they quit smoking at the time of cancer diagnosis, 13–60% they restart when treatment is finished [5].

Although the short and long-term adverse effects of HTPs are not clear, tobacco companies have been marketing them to smokers as better alternatives to conventional cigarettes based on assertions that they expose users to lower levels of toxicants. It has been shown that advertisements and TV programs impacted on interest in the new type of tobacco products, such as HTPs and e-cigarettes [6,7], and awareness of HTPs has been increasing recently in Japan [8,9]. Popova et al also revealed that qualitative and quantitative studies conducted by PMI demonstrate that adult consumers perceive reduced exposure claims as reduced risk claims [10]. These promotions and mass media campaigns may encourage people with chronic disease to start using HTPs as a way to improve their health.

People with chronic disease have difficulty in stopping smoking, but there is no evidence about the use of HTPs or dual use with conventional cigarettes in people with chronic disease. People with chronic disease may be more likely to use HTPs and both HTPs and cigarettes together than those without the disease for their health. The aim of this study is to reveal the use of HTPs in people with chronic disease and we cross-sectionally investigate the association of chronic disease with current use of HTPs and dual use with conventional cigarettes.

## Materials and methods

The study was reviewed and approved by the Research Ethics Committee of the Osaka International Cancer Institute (no. 1412175183) and the National Institute of Public Health (NIPH-IBRA#12112)".

### Participants

The Japan “Society and New Tobacco” Internet Survey (JASTIS) study was a longitudinal internet study designed to investigate the prevalence of HTP and e-cigarette use and the ways to use tobacco products. It also recorded information on demographics, socioeconomic status, and health conditions from 2015. Detailed information on JASTIS is provided in the study profile paper [11]. Briefly, participants were recruited from a large survey panel managed by a major internet research agency, Rakuten Insight (formerly Rakuten Research), which maintains a pool of 2.3 million panelists in Japan [12]. At the time of registration, the panelists agree to participate in research surveys with web-based written consent. Minors provided their consent with approval from their parents or guardians.

**Fig 1:**
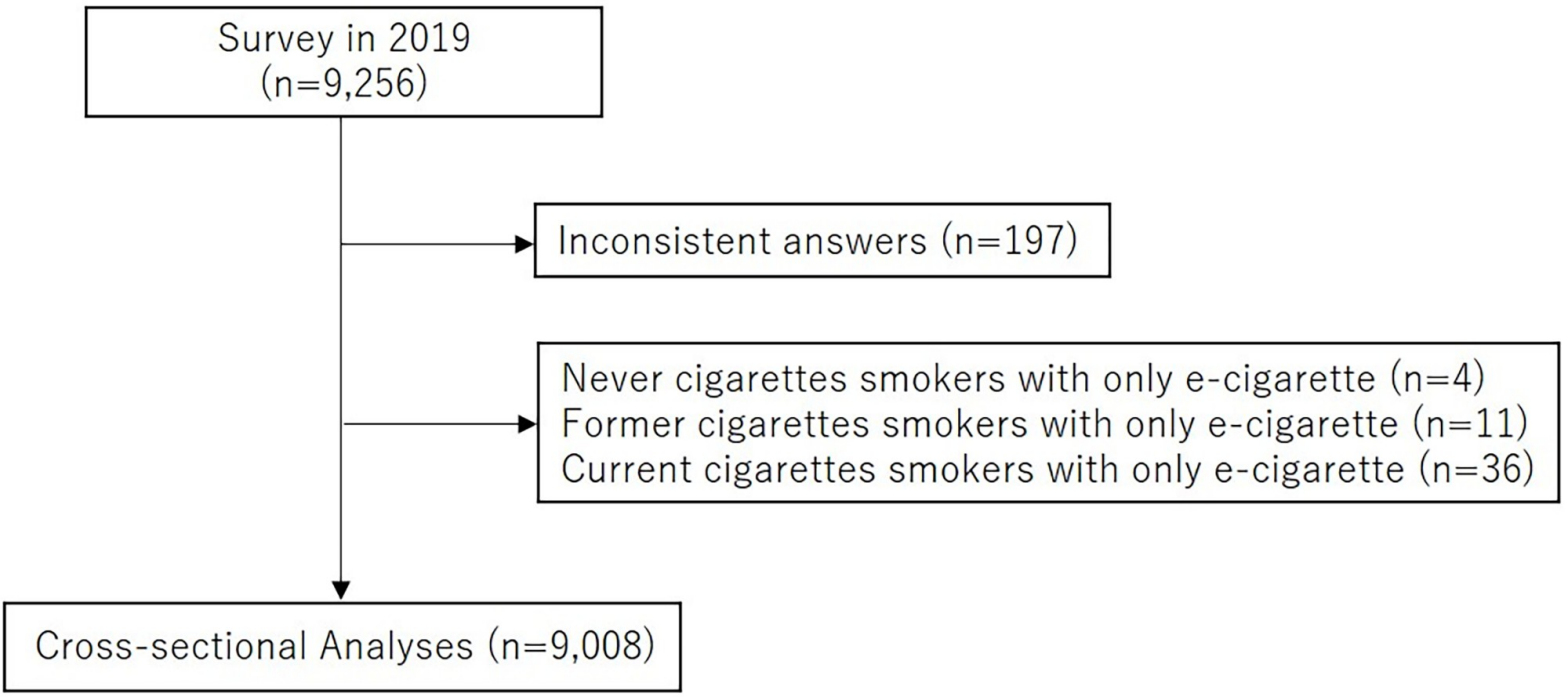
Flow diagram of study selection strategy.

We cross-sectionally analyzed 9,256 participants who were randomly recruited based on sex and age (men and women aged 15–69 years at baseline in 2015) and answered the questionnaires in February 2019. We excluded 197 people with inconsistent answers, and 4 never cigarettes smokers, 11 former cigarettes smokers, and 36 current cigarettes smokers with only e-cigarette use in the previous 30 days, because, in this study, we did not pay attention to e-cigarettes, which are rarely used in Japan. We finally analyzed 9,008 participants (4,414 men and 4,594 women) (Fig 1).

### HTPs and cigarettes use

HTPs in this study included Ploom TECH (Japan Tobacco), IQOS (Philip Morris International), and glo (British American Tobacco), which were available in Japan on the date of the survey. The following definitions were used: ‘HTP use’, HTP use including exclusive HTP use and dual use in the previous 30 days; ‘current cigarettes smoker’, a person who had used conventional cigarettes in the previous 30 days; ‘former cigarettes smoker’, a person who used to smoke, and ‘never cigarettes smoker’ a person who had never smoked regularly. Participants were asked about their use of cigarettes and each HTP (Ploom, IQOS, and glo) in the previous 30 days using the following question: “Have you used the following products in the previous 30 days? [6]”

### Other variables

We recruited participants aged 15–69 years at baseline and classified them into six groups by age in 2019; 15–19, 20–29, 30–39, 40–49, 50–59, 60–73 years old. Equivalent household income was obtained using internet questionnaires and we divided the population into quartiles and a ‘did not know /unwilling to answer’ group. The 25th, median, and 75th equivalent household income of the study population were 2250.0, 3250.0, and 4907.5 thousand-yen. Education status was classified into three groups; junior high school, high school, or other, technical school or college, and university or graduate school. Drinking status was divided three groups: never, former, current drinker. Information on medical histories including hypertension, diabetes, angina, MI, stroke, asthma, chronic obstructive pulmonary disease (COPD) and cancer, was obtained from the questionnaires. We defined cardiovascular disease (CVD) as having at least one of angina, MI, and stroke.

### Statistical analysis

Participant characteristics were reported as the number and percentage of participants by categories. Predictors for dual use with cigarettes among HTP users were analyzed using the chi-square test or Fishers’ exact test. We used logistic regressions to calculate the odds ratios (ORs) and 95% confidence intervals (CIs) of medical histories for the use of HTPs, cigarettes, both HTPs and cigarettes, or not using HTPs or cigarettes. For multivariable adjustments in logistic regressions, we first adjusted for age, sex, and cigarettes or HTPs if necessary (Model 2), then further adjusted for equivalent household income, education, and drinking status (Model 3). Two-tailed p-values of <0.05 were considered to indicate statistical significance. All statistical analyses were performed with SAS software (version 9.4, SAS Institute, Cary, North Carolina).

## Results

Participants comprised 852 panelists aged 15–19 years, 1,545 aged 20–29 years, 1,409 aged 30–39 years, 1,694 aged 40–49 years, 1,564 aged 50–59 years, and 1,944 aged 60–73 years. Of these, 4,414 were males and 4,594 females (Table 1). The prevalence of current HTP use including dual use and dual use with cigarettes was 9.0% and 6.1% respectively in total. By disease: 10.2% and 7.4% for hypertension, 15.9% and 12.3% for diabetes, 19.2% and 15.7% for CVD, 40.5% and 33.3% for COPD, and 17.5% and 11.9% for cancer. The number of people who use each type of HTP were: 445 for Ploom TECH, 443 for IQOS, and 265 for glo. Also, 213 people (10.2%) among 2,099 former smokers use HTPs, while 546 people (41.7%) of 1,309 current smokers use HTPs; i.e., current smokers are significantly likely to use HTPs than former smokers (p<0.0001).

**Table 1:** Characteristics of participants.

	Never cigarettes smoker		Former cigarettes smoker		Current cigarettes smoker		Total
	HTPs use (-)	HTPs use (+)	HTPs use (-)	HTPs use (+)	HTPs use (-)	HTPs use (+)	
Number	5,546 (61.57%)	54 (0.60%)	1,886 (20.94%)	213 (2.36%)	763 (8.47%)	546 (6.06%)	9,008
Age (years)							
15–19	797 (93.54%)	8 (0.94%)	9 (1.06%)	1 (0.12%)	14 (1.64%)	23 (2.70%)	852
20–29	1,237 (80.06%)	26(1.68%)	80 (5.18%)	27 (1.75%)	67 (4.34%)	108 (6.99%)	1,545
30–39	879 (62.38%)	10(0.71%)	254 (18.03%)	54 (3.83%)	98 (6.96%)	114 (8.09%)	1,409
40–49	918 (54.19%)	5(0.30%)	400 (23.61%)	56 (3.31%)	186 (10.98%)	129 (7.62%)	1,694
50–59	773 (49.42%)	5(0.32%)	436 (27.88%)	51 (3.26%)	206 (13.17%)	93 (5.95%)	1,564
60–73	942 (48.46%)	0(0%)	707 (36.37%)	24 (1.23%)	192 (9.88%)	79 (4.06%)	1,944
Sex							
Male	1,996 (45.22%)	26 (0.59%)	1284 (29.09%)	156 (3.53%)	539 (12.21%)	413 (9.36%)	4,414
Female	3,550 (77.27%)	28 (0.61%)	602 (13.10%)	57 (1.24%)	224 (4.88%)	133 (2.90%)	4,594
Equivalent household income							
1^st^ quartile (Lowest)	1,161 (64.82%)	10 (0.56%)	334 (18.65%)	36 (2.01%)	166 (9.27%)	84 (4.69%)	1,791
2^nd^ quartile	1,026 (59.89%)	18 (1.05%)	403 (23.53%)	39 (2.28%)	134 (7.82%)	93 (5.43%)	1,713
3^rd^ quartile	998 (56.64%)	7 (0.40%)	401 (22.76%)	47 (2.67%)	161 (9.14%)	148 (8.40%)	1,762
4^th^ quartile (Highest)	932 (53.97%)	11 (0.64%)	434 (25.13%)	56 (3.24%)	152 (8.80%)	142 (8.22%)	1,727
Did not know / Unwilling to answer	1,429 (70.92%)	8 (0.40%)	314 (15.58%)	35 (1.74%)	150 (7.44%)	79 (3.92%)	2,015
Education							
Junior high school / High school / Other	1,872 (63.41%)	24 (0.81%)	530 (17.95%)	72 (2.44%)	282 (9.55%)	172 (5.83%)	2,952
Technical school / College	1,141 (63.35%)	9 (0.50%)	367 (20.38%)	41 (2.28%)	159 (8.83%)	84 (4.66%)	1,801
University / Graduate school	2,533 (59.53%)	21 (0.49%)	989 (23.24%)	100 (2.35%)	322 (7.57%)	290 (6.82%)	4,255
Drinking							
Never	2,339 (77.60%)	14 (0.46%)	380 (12.61%)	50 (1.66%)	160 (5.31%)	71 (2.36%)	3,014
Former	708 (66.35%)	2 (0.19%)	211 (19.78%)	20 (1.87%)	72 (6.75%)	54 (5.06%)	1,067
Current	2,499 (50.72%)	38 (0.77%)	1295 (26.28%)	143 (2.90%)	531 (10.78%)	421 (8.54%)	4,927
Type of HTP							
Ploom TECH		11 (2.47%)		88 (19.78%)		346 (77.75%)	445
IQOS		43 (9.71%)		126 (28.44%)		274 (61.85%)	443
Glo		12 (4.53%)		73 (27.55%)		180 (67.92%)	265
Hypertension	543 (43.41%)	1 (0.08%)	458 (36.61%)	34 (2.72%)	123 (9.83%)	92 (7.35%)	1251
Diabetes	151 (35.78%)	1 (0.24%)	148 (35.07%)	14 (3.32%)	56 (13.27%)	52 (12.32%)	422
Angina	37 (38.54%)	2 (2.08%)	30 (31.25%)	1 (1.04%)	7 (7.29%)	19 (19.79%)	96
Myocardial Infarction	15 (27.27%)	2 (3.64%)	20 (36.36%)	0 (0%)	4 (7.27%)	14 (25.45%)	55
Stroke	29 (41.43%)	0 (0%)	22 (31.43%)	3 (4.29%)	3 (4.29%)	13 (18.57%)	70
CVD	68 (39.53%)	2 (1.16%)	61 (35.47%)	4 (2.33%)	10 (5.81%)	27 (15.70%)	172
Asthma	277 (65.18%)	4 (0.94%)	75 (17.65%)	10 (2.35%)	21 (4.94%)	38 (8.94%)	425
COPD	10 (23.81%)	0 (0%)	10 (23.81%)	3 (7.14%)	5 (11.90%)	14 (33.33%)	42
Cancer	62 (49.21%)	1 (0.79%)	40 (31.75%)	6 (4.76%)	2 (1.59%)	15 (11.90%)	126

Equivalent household income: 25^th^ = 2250.0 thousand-yen, 50^th^ = 3250.0 thousand-yen, 75^th^ = 4907.5 thousand-yen, COPD: Chronic obstructive pulmonary disease, CVD: Cardiovascular disease, HTP: Heated tobacco product.

Analysis of the predictors for HTPs and cigarettes dual use among all HTP users is shown in Table 2. Sex and drinking status were significantly related to dual use with cigarettes (p = 0.02 and p<0.001, respectively). Patients with diabetes, angina, and CVD were tend to be use cigarettes with HTPs than people without each disease (p = 0.06, p = 0.06, and p = 0.09, respectively). Age, income, and education were not related to dual use with cigarettes.

**Table 2:** Factors for dual use with cigarettes among all HTP users.

	Total	Only HTPs use	HTPs and cigarettes dual use	p-value
Number	813	267 (32.84%)	546 (67.16%)	
Age (years)				0.23
15–19	32	9 (28.13%)	23 (71.88%)	
20–29	161	53 (32.92%)	108 (67.08%)	
30–39	178	64 (35.96%)	114 (64.04%)	
40–49	190	61 (32.11%)	129 (67.89%)	
50–59	149	56 (37.58%)	93 (62.42%)	
60–73	103	24 (23.30%)	79 (76.70%)	
Sex				0.02
Male	595	182 (30.59%)	413 (69.41%)	
Female	218	85 (38.99%)	133 (61.01%)	
Equivalent household income				0.20
1^st^ quartile (Lowest)	130	46 (35.38%)	84 (64.62%)	
2^nd^ quartile	150	57 (38.00%)	93 (62.00%)	
3^rd^ quartile	202	54 (26.73%)	148 (73.27%)	
4^th^ quartile (Highest)	209	67 (32.06%)	142 (67.94%)	
Did not know / Unwilling to answer	122	43 (35.25%)	79 (64.75%)	
Education				0.11
Junior high school / High school / Other	268	96 (35.82%)	172 (64.18%)	
Technical school / College	134	50 (37.31%)	84 (62.69%)	
University / Graduate school	411	121 (29.44%)	290 (70.56%)	
Drinking				<0.001
Never	135	64 (47.41%)	71 (52.59%)	
Former	76	22 (28.95%)	54 (71.05%)	
Current	602	181 (30.07%)	421 (69.93%)	
Hypertension				0.17
No	686	232 (33.82%)	454 (66.18%)	
Yes	127	35 (27.56%)	92 (72.44%)	
Diabetes				0.06
No	746	252 (33.78%)	494 (66.22%)	
Yes	67	15 (22.39%)	52 (77.61%)	
Angina				0.06
No	791	264 (33.38%)	527 (66.62%)	
Yes	22	3 (13.64%)	19 (86.36%)	
Myocardial Infarction				0.11
No	797	265 (33.25%)	532 (66.75%)	
Yes	16	2 (12.50%)	14 (87.50%)	
Stroke				0.29
No	797	264 (33.12%)	533 (66.88%)	
Yes	16	3 (18.75%)	13 (81.25%)	
CVD				0.09
No	780	261 (33.46%)	519 (66.54%)	
Yes	33	6 (18.18%)	27 (81.82%)	
Asthma				0.35
No	761	253 (33.25%)	508 (66.75%)	
Yes	52	14 (26.92%)	38 (73.08%)	
COPD				0.20
No	796	264 (33.17%)	532 (66.83%)	
Yes	17	3 (17.65%)	14 (82.35%)	
Cancer				1.00
No	791	260 (32.87%)	531 (67.13%)	
Yes	22	7 (31.82%)	15 (68.18%)	

Equivalent household income: 25^th^ = 2250.0 thousand-yen, 50^th^ = 3250.0 thousand-yen, 75^th^ = 4907.5 thousand-yen, COPD: Chronic obstructive pulmonary disease, CVD: Cardiovascular disease, HTP: Heated tobacco product.

**Fig 2:**
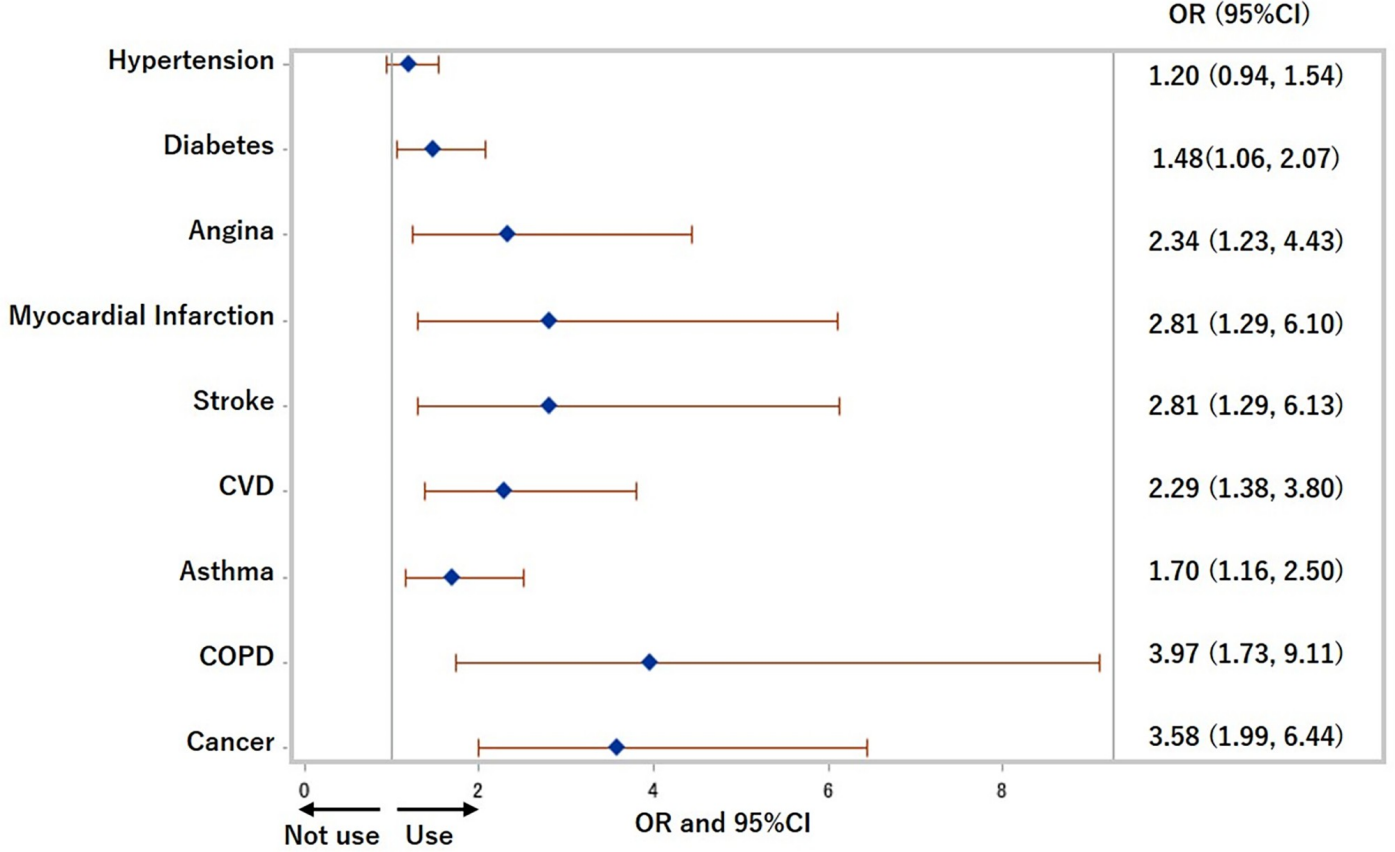
Odds ratios for use of heated tobacco products in previous 30 days. COPD: Chronic obstructive pulmonary disease, CVD: Cardiovascular disease, OR: Odds ratio. Adjusted for age, sex, cigarettes use, equivalent household income, education, and drinking status.

**Fig 3:**
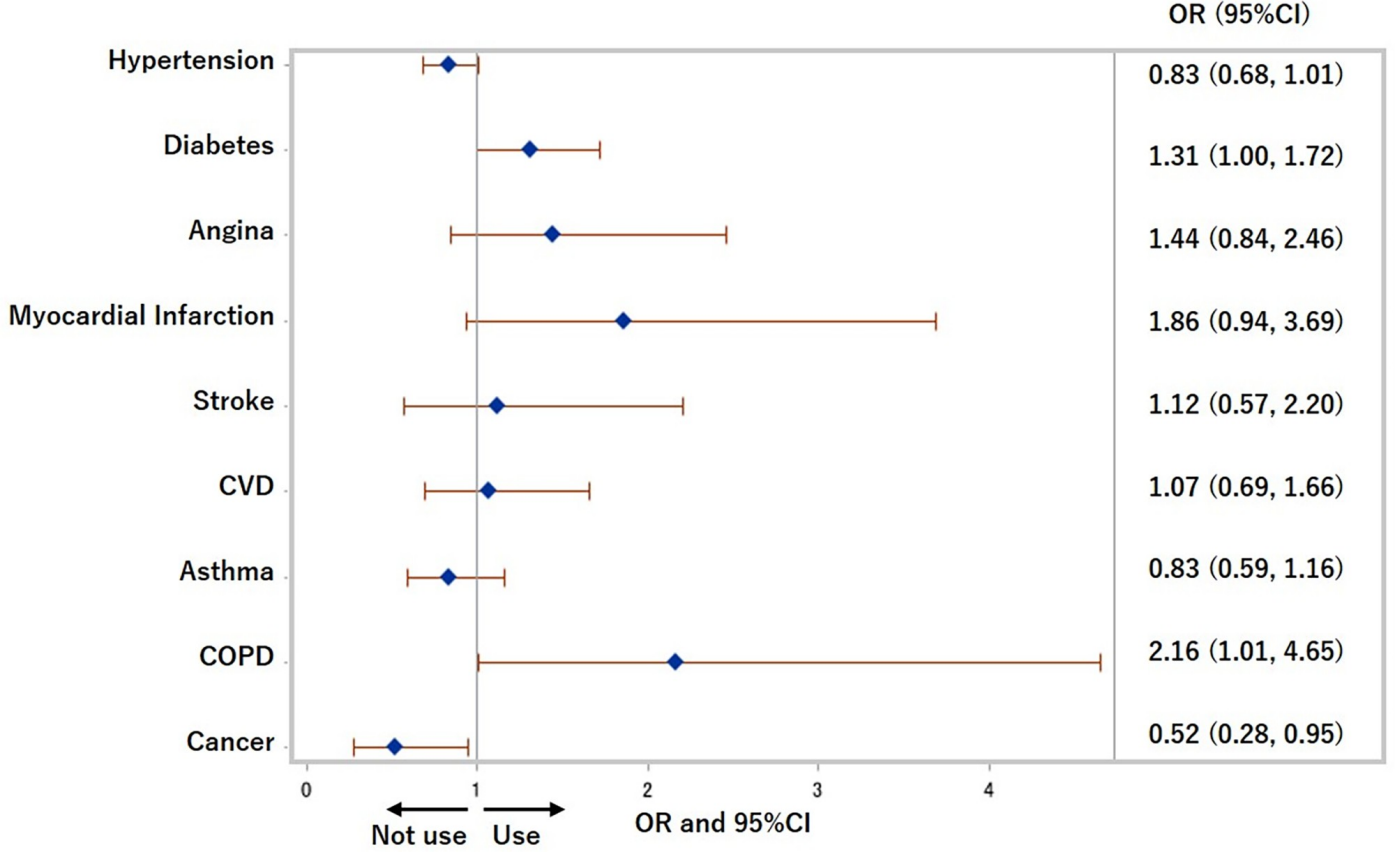
Odds ratios for use of cigarettes in previous 30 days. COPD: Chronic obstructive pulmonary disease, CVD: Cardiovascular disease, OR: Odds ratio. Adjusted for age, sex, heated tobacco products use, equivalent household income, education, and drinking status.

The ORs and 95% CIs of each chronic disease for HTP use within the previous 30 days were 1.48 (1.06, 2.07) (p = 0.02) for diabetes, 2.29 (1.38, 3.80) (p<0.01) for CVD, 1.70 (1.16, 2.50) (p<0.01) for asthma, 3.97 (1.73, 9.11) (p<0.01) for COPD, and 3.58 (1.99, 6.44) (p<0.0001) for cancer after adjusting for age, sex, cigarettes use, equivalent household income, education, and drinking status in Model 3 (Fig 2). Also, people with cancer were less likely to use cigarettes (OR: 0.52 (0.28, 0.95), p = 0.03), and people with COPD were more likely to use cigarettes (OR: 2.16 (1.01, 4.65), p = 0.05) after adjusting for age, sex, HTPs use, equivalent household income, education, and drinking status in Model 3 (Fig 3).

**Fig 4:**
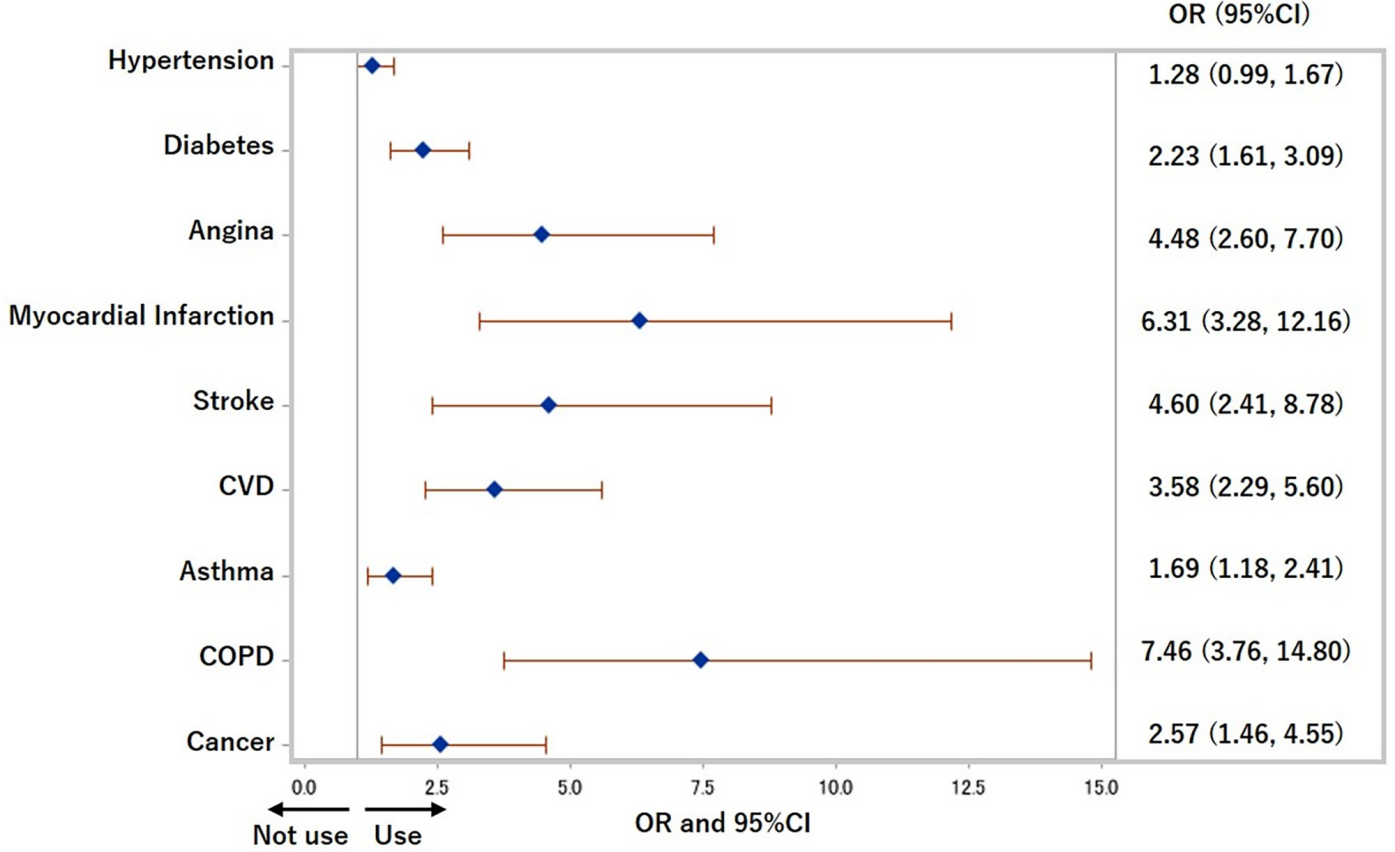
Odds ratios for use of both cigarettes and heated tobacco products in previous 30 days. COPD: Chronic obstructive pulmonary disease, CVD: Cardiovascular disease, OR: Odds ratio. Adjusted for age, sex, equivalent household income, education, and drinking status.

**Fig 5:**
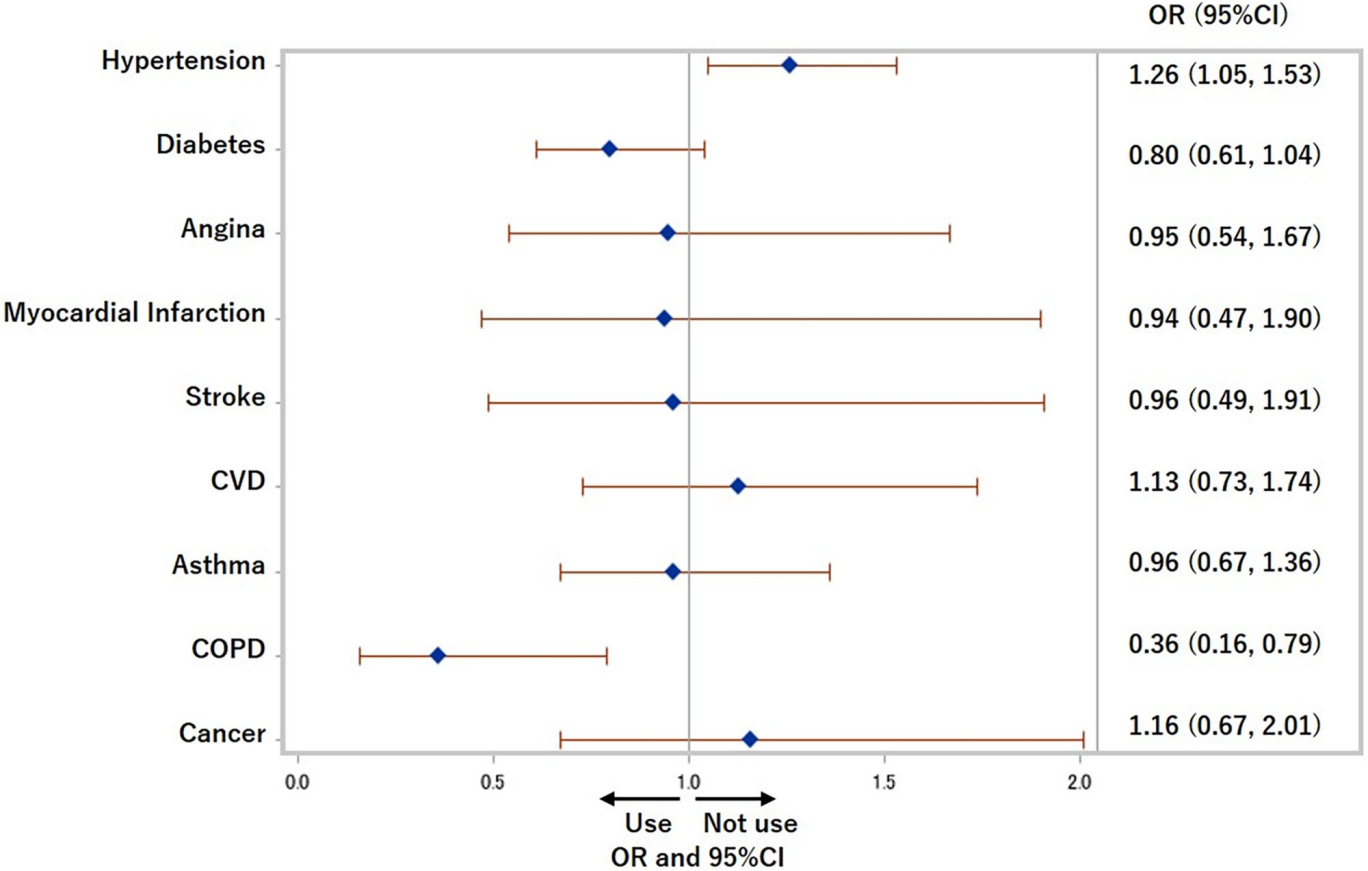
Odds ratios for not using cigarettes nor heated tobacco products in previous 30 days among former and current cigarettes smokers. COPD: Chronic obstructive pulmonary disease, CVD: Cardiovascular disease, OR: Odds ratio. Adjusted for age, sex, equivalent household income, education, and drinking status.

The ORs and 95% CIs of each chronic disease for the use of both cigarettes and HTPs were 2.23 (1.61, 3.09) (p<0.0001) for diabetes, 3.58 (2.29, 5.60) (p<0.0001) for CVD, 1.69 (1.18, 2.41) (p<0.01) for asthma, 7.46 (3.76, 14.80) (p<0.0001) for COPD, and 2.57 (1.46, 4.55) (p<0.0001) for cancer after adjusting for traditional confounders in Model 3 (Fig 4). The ORs and 95% CIs of each medical history for not using cigarettes or HTPs in the previous 30 days among former and current cigarettes smokers are shown in Fig 5. People with hypertension were more likely not to use cigarettes or HTPs (OR: 1.26 (1.05, 1.53), p = 0.02), and people with COPD were less likely not to use cigarettes or HTPs (more likely to use at least one of cigarettes and HTPs) (OR: 0.36 (0.16, 0.79), p = 0.01). CVD, asthma, and cancer were not related to not using cigarettes or HTPs.

## Discussion

To the best of our knowledge, this is the first study to reveal that people with chronic diseases, such as CVD, asthma, COPD, and cancer, are more likely to use HTPs and both HTPs and cigarettes together than those without the disease.

### Health effect of HTPs

HTPs have appeared on the market relatively recently with the claim by tobacco companies that they have lower levels of toxicants and are less dangerous than conventional cigarettes. However, aerosols from HTPs include nicotine, some carcinogens, additives, and flavored substances, that might impact on health [13–15], and the levels of some toxins in HTPs are higher than in cigarettes [16]. St Helen et al found that PMI reported levels for only 40 of 93 harmful and potentially harmful constituents (HPHCs) identified by the FDA in IQOS mainstream aerosol [17]. PMI’s data also showed significantly higher levels of many toxicants not on the FDA HPHC list in IQOS aerosol compared with cigarette smoke. In some reports about carcinogens in HTP aerosol, it is reported formaldehyde was 1–74%, and acetaldehyde was 1–22% compared with reference cigarettes [13,18,19]. However, the number of these chemicals is almost same as conventional cigarettes. Moreover, the carcinogens have no threshold for cancers, and even small amounts of these chemicals might affect the health of the individual user. Also, aerosols from HTPs include 13–84% nicotine compared with reference cigarettes, which is high enough to induce addiction [13,18,19]. In addition to tobacco components, glycerin, propylene glycol, and triacetin are added to HTPs. These additives release formaldehyde, acetone, and acetaldehyde, all of which are carcinogens when they are heated [20]. Flavored ingredients (menthol, etc.) and metals from the devices (chromium, nickel, and lead, etc.) might be also toxic [21–23].

Adverse effects of HTPs for cardiovascular risk and lung function are reported. Nabavizadeh et al showed IQOS aerosol’s acute effects impaired vascular endothelial function measured with Flow-Mediated Dilatation, which is known to be impaired by conventional cigarette [24]. Further, PMI study included measurement of a broad panel of biomarkers associated with cardiovascular risk, and most of the cardiovascular biomarkers did not change after switching to HTPs [25,26]. These observations suggest that switching to HTPs is not likely to reduce the risk of cardiovascular morbidity and mortality associated with conventional cigarettes [27]. HTPs also cause cytotoxic effects for human bronchial epithelium [28], and a case of acute eosinophilic pneumonia following HTPs smoking has been reported [29]. Recently, the FDA has denied the risk-reduced claim made for IQOS [30].

### Smoking cessation using e-cigarettes and HTPs and the risks of dual use with cigarettes

The impact of HTPs on smoking cessation is unknown. While some epidemiological studies and clinical trials have shown that e-cigarettes are effective for smoking cessation [31–33], other epidemiological and meta-analyses, including clinical studies, have shown the opposite [1,34–36]. One Japanese cross-sectional study showed that, for effective smoking cessation, e-cigarette use appears to have low efficacy among smokers [37]. Also, HTPs are sometimes used with conventional cigarettes. In our study, the prevalence of dual use of HTPs with cigarettes was 6.1% in the total study sample, 12.3% for diabetes, 15.7% for CVD, 33.3% for COPD, 11.9% for cancer, and 41.7% for current smokers. It has been shown that e-cigarettes, which contain smaller amounts of chemicals, do not reduce exposure to toxic chemicals when they are used with conventional cigarettes [38]. Reddy et al reported that dual use of e-cigarettes with combustible tobacco is significantly associated with incident respiratory symptoms compared with either product alone [39]. Dual use of e-cigarettes with conventional cigarettes were also related to greater nicotine dependence [40]. Similarly, HTPs would not reduce the amounts of chemicals emitted when they are used with conventional cigarettes. It is important to address the problem of how to encourage dual users to stop smoking.

### Adverse effect of tobacco in chronic disease

The causal relationship between conventional cigarettes and the prognosis of chronic diseases is well known. It has been reported that following MI, the recurrence rate and the rate of death in patients who quit smoking was less than that of patients who continue smoking [41,42]. In COPD patients, the decrease rate of forced expiratory volume in 1 second as a percentage of FVC (FEV1.0%) in patients who quit smoking was about half that in patients who continued to smoke [43]. Smoking cessation after a diagnosis of cancer decreases the risk of recurrence and secondary cancers, alleviates cancer treatment complications and reduces overall mortality [5]. Since HTPs might cause the same adverse events as conventional cigarettes, people should quit smoking both cigarettes and HTPs, which include nicotine, carcinogens, and additives, in order to prevent the incidence and recurrence of tobacco-related diseases.

### Smoking situation of chronic disease patients

Many patients cannot stop smoking even if they have chronic diseases. For example, it has been reported that only a third to a half of the smokers after MI reduce or quit smoking [4], and a half of patients who quit smoking after coronary artery bypass grafting or percutaneous coronary intervention, start again within 1 year [44]. Also, a review found that the self-reported prevalence of smoking among cancer survivors ranges between 9% and 33%, and even if patients with cancer quit smoking at the time of diagnosis, 13–60% restart after treatment is over and with time, smoking abstinence rates among cancer survivors gradually decrease [5]. In this study, people with cancer were less likely to use cigarettes than people without cancer. This may indicate that the onset of cancer may be a teachable moment to quit smoking, but all people with cancer cannot stop smoking in this study. On the other hand, people with COPD were more likely to use cigarettes in this study. The Behavioral Risk Factor Surveillance System in the United States showed that current smoking rate in patients with COPD was 45.1%, and it was higher than that observed among adults with other chronic disease (23%), asthma (20.3%), and no chronic disease (18.9%) [45]. The main cause of COPD is smoking, and there may be strong nicotine dependence in COPD patients. Again, appropriate tobacco cessation support may be needed for patients with chronic disease.

### Limitations

There were some limitations to this study. First, the data was obtained through a self-reported internet survey, and the information on the medical history was also acquired from self-reported questionnaires, not medical records. However, we excluded respondents with discrepancies or inconsistencies in their answer to improve the accuracy of the study. Second, the number of people with specific medical histories, especially COPD, was relatively low. Thus, this may result in non-significant association between chronic disease and current use of HTPs and dual use with cigarettes, although some significant association was observed in COPD patients. Finally, our data are limited to the Japanese population. However, the problem of smoking cessation in people with chronic disease is the same all over the world, and the desire of chronically ill patients to stop smoking is also similar.

## Conclusions

In conclusion, we found that people with chronic disease, such as CVD, asthma, COPD, and cancer, were more likely to use HTPs or HTPs and cigarettes together. We believe that these findings mean that people with chronic disease start to use HTPs for their health but, in reality, continue to use HTPs or cigarettes and HTPs together. We can surmise that people with chronic disease find it difficult to stop smoking, and further research on the smoking situation of HTPs in patients with chronic diseases is necessary.
